# How Does the Sweet Violet (*Viola odorata* L.) Fight Pathogens and Pests – Cyclotides as a Comprehensive Plant Host Defense System

**DOI:** 10.3389/fpls.2018.01296

**Published:** 2018-09-11

**Authors:** Blazej Slazak, Małgorzata Kapusta, Adam A. Strömstedt, Aneta Słomka, Marta Krychowiak, Mohammadreza Shariatgorji, Per E. Andrén, Jerzy Bohdanowicz, Elżbieta Kuta, Ulf Göransson

**Affiliations:** ^1^W. Szafer Institute of Botany, Polish Academy of Sciences, Kraków, Poland; ^2^Pharmacognosy, Department of Medicinal Chemistry, Uppsala University, Uppsala, Sweden; ^3^Department of Plant Cytology and Embryology, Faculty of Biology, University of Gdańsk, Gdańsk, Poland; ^4^Department of Plant Cytology and Embryology, Institute of Botany, Jagiellonian University, Kraków, Poland; ^5^Laboratory of Biologically Active Compounds, Intercollegiate Faculty of Biotechnology, University of Gdańsk and Medical University of Gdańsk, Gdańsk, Poland; ^6^Medical Mass Spectrometry Imaging, National Resource for Mass Spectrometry Imaging, Science for Life Laboratory, Department of Pharmaceutical Biosciences, Uppsala University, Uppsala, Sweden

**Keywords:** cyclotides, plant host defense, Violaceae, antimicrobial peptide, antifungal defense, MALDI-MSI, immunohistochemistry

## Abstract

Cyclotides are cyclic plant polypeptides of 27–37 amino acid residues. They have been extensively studied in bioengineering and drug development contexts. However, less is known about the relevance of cyclotides for the plants producing them. The anti-insect larvae effects of kB1 and antibacterial activity of cyO2 suggest that cyclotides are a part of plant host defense. The sweet violet (*Viola odorata* L.) produces a wide array of cyclotides, including kB1 (kalata B1) and cyO2 (cycloviolacin O2), with distinct presumed biological roles. Here, we evaluate V. odorata cyclotides’ potency against plant pathogens and their mode of action using bioassays, liposome experiments and immunogold labeling for transmission electron microscopy (TEM). We explore the link between the biological activity and distribution in plant generative, vegetative tissues and seeds, depicted by immunohistochemistry and matrix assisted laser desorption ionization mass spectrometry imaging (MALDI-MSI). Cyclotides cyO2, cyO3, cyO13, and cyO19 are shown to have potent activity against model fungal plant pathogens (*Fusarium oxysporum, F. graminearum, F. culmorum, Mycosphaerella fragariae, Botrytis cinerea*) and fungi isolated from violets (*Colletotrichum utrechtense and Alternaria alternata*), with minimal inhibitory concentrations (MICs) ranging from 0.8 μM to 25 μM. Inhibition of phytopathogenic bacteria – *Pseudomonas syringae* pv. *syringae, Dickeya dadantii* and *Pectobacterium atrosepticum* – is also observed with MIC = 25–100 μM. A membrane-disrupting antifungal mode of action is shown. Finding cyO2 inside the fungal spore cells in TEM images may indicate that other, intracellular targets may be involved in the mechanism of toxicity. Fungi can not break down cyclotides in the course of days. varv A (kalata S) and kB1 show little potency against pathogenic fungi when compared with the tested cycloviolacins. cyO2, cyO3, cyO19 and kB1 are differentially distributed and found in tissues vulnerable to pathogen (epidermis, rizodermis, vascular bundles, protodermis, procambium, ovary walls, outer integuments) and pest (ground tissues of leaf and petiole) attacks, respectively, indicating a link between the cyclotides’ sites of accumulation and biological role. Cyclotides emerge as a comprehensive defense system in *V. odorata*, in which different types of peptides have specific targets that determine their distribution in plant tissues.

## Introduction

The polypeptides now known as cyclotides were first reported in the 1970s when Lorents Gran isolated kalata B1 (kB1) from *Oldenlandia affinis* (Rubiaceae) (Gran, 1973). In the mid 90s, NMR spectroscopic analysis of kB1 revealed the circular peptide backbone of about 30 amino acid residues and the cystine knot that are the structural hallmarks of the cyclotides (Saether et al., 1995). Later discoveries established them as a family of polypeptides, and the term cyclotides (*cyclo*-pep*tides*) was coined (Craik et al., 1999). The cyclotides can be divided into two subfamilies (the Möbius and bracelet), which are distinguished by the presence and absence, respectively, of a *cis* proline in loop 5 between cysteines 5 and 6 (Rosengren et al., 2003). Today they are recognized as the largest known circular polypeptide families (Burman et al., 2014). Cyclotide-producing plants accumulate large amounts of these peptides—up to 1.5 g per kg of wet mass (Ireland et al., 2006). Producing a wide diversity of cyclotides in such large quantities must be costly to the plant, which raises the question of how does the plant benefit from this expenditure.

Cyclotides are synthesized and deposited in all parts of the plants producing them, but individual organs and tissues have their own distinct cyclotide distributions that differ in terms of quantity and diversity of sequences (Trabi and Craik, 2004; Slazak et al., 2015). It is suggested that the cyclotide suites expressed in specific organs and tissues are shaped by the environment and the challenges it presents to the plant, such as pathogen or insect attacks (Gilding et al., 2016; Slazak et al., 2016). Therefore, the distribution of particular peptides is probably linked to their biological role.

Cyclotides have been found in six angiosperm families: Rubiaceae, Cucurbitaceae, Fabaceae, Solanaceae, Poaceae, and Violaceae (Gran, 1973; Göransson et al., 1999; Hernandez et al., 2000; Poth et al., 2011, 2012; Nguyen et al., 2013; Burman et al., 2015). The Violaceae is particularly interesting because each member of this family appears to produce cyclotides (Burman et al., 2015). In some instances, species belonging to distantly related plant families express cyclotides with identical sequences and structures. For example, varv A (synonym kalata S) and the prototypical cyclotide kB1 are found in both, violets (Violaceae) and *Oldenlandia affinis* (Rubiaceae) (Gran, 1973; Claeson et al., 1998; Göransson et al., 1999; Ireland et al., 2006). *V. odorata*, belonging to the Violaceae, has become a good model species in cyclotide research. This plant express both bracelet cycloviolacins (e.g., cycloviolacin O2, cyO2) and Möbius kalata (e.g., kB1) cyclotides that have distinct biological activities and thus presumably different roles (Craik et al., 1999; Ireland et al., 2006).

Cyclotides have been shown to exhibit diverse biological activities including uterotonic, hemolytic, anti-HIV, cytotoxic, and antimicrobial (Jennings et al., 2001; Svangård et al., 2007; Wang et al., 2008; Pränting et al., 2010). Most of these activities are associated with their ability to selectively bind to certain lipids and to disrupt lipid membranes (Henriques et al., 2011; Strömstedt et al., 2017).

Although the structure and biological activity of cyclotides have been studied extensively for the purposes of peptide engineering and to assess their potential in drug discovery (Weidmann and Craik, 2016), less is known about their role in the plants that produce them. The most popular hypothesis is that cyclotides are constituents of a plant defense system. This hypothesis is largely based on the anti-insect larvae effects of Möbius kalata peptides (kB1, kB2) produced by *Oldenlandia affinis* (Rubiaceae) (Jennings et al., 2001, 2005). However, the biological role of bracelet cycloviolacin cyclotides produced by violets and all other members of the Violaceae is poorly understood. Based on immunohistochemistry, we recently suggested that they also have a role in defense mechanisms: it was found that cyclotides in *V. odorata* and *V. uliginosa* accumulate in tissues vulnerable to microbial pathogens and pests, such as the epidermis and vascular bundles (Slazak et al., 2016). Cycloviolacin cyclotides have also been found to be active against human pathogens including *Candida* (Tam et al., 1999; Strömstedt et al., 2017) and bacteria, particularly Gram-negative species (Pränting et al., 2010; Strömstedt et al., 2017). However, the importance of cyclotides’ antimicrobial activity has not yet been studied in phytopathogenic contexts, using methods that reflect the specific conditions present in plant tissues.

This report describes a comprehensive analysis of the central role played by *V. odorata* cyclotides in defense mechanisms. We investigate the activity of cycloviolacin cyclotides from *V. odorata* and *V. uliginosa* (cyO2, cyO3, cyO13, cyO19) against plant pathogens and describe their antifungal mode of action. We also demonstrate a connection between cyclotides’ biological role and their distribution in generative and vegetative tissues.

## Materials and Methods

### Plant Material

Dried aerial parts of *Viola odorata* L. and *Viola tricolor* L. were bought from Galke (Gittelde, Germany) for bulk extraction, and living *V. odorata* L. specimens were collected by EK in Cracow-Ugorek (Poland) during the summer of 2016. *V. odorata* flowers and seeds were collected by AS and EK from the same locality as above during the summer 2015. A callus suspension culture of *V. uliginosa* Besser derived from leaves was obtained as previously described (Slazak et al., 2015).

### Cyclotide Extraction

Cyclotides cyO2, cyO3, cyO19, varv A (kalata S), kB1 were extracted with 60% methanol in water from *V. odorata* and *V. tricolor* dried plant material as described previously (Göransson et al., 2004). To obtain cyO13, approximately 3 g of freeze-dried *V. uliginosa* suspension culture material, acquired as described previously (Slazak et al., 2015), was extracted three times with 30% acetonitrile (ACN) and 0.05% trifluoroacetic acid (TFA) in water. C18 Isolute^®^ C18 SPE columns (Argonaut Technologies) were used to purify the cyclotide fraction: the extract was diluted 3× with water, loaded onto the column, and washed with 10% aq. ACN, 0.05% TFA (buffer A) to elute hydrophilic compounds, after which the cyclotide-containing fraction was eluted with 60% ACN and 0.05% TFA in water (buffer B), collected, freeze-dried, and reconstituted in buffer A. Peptides were purified through HPLC as described by Herrmann et al. (2008) to a purity not less than 95%.

Concentrations of stock peptide solutions were determined using a NanoDrop 2000c instrument (Thermo Scientific, Waltham, MA, United States) based on their absorbance at 280 nm. The measurements were adjusted based on the cyclotides’ molecular masses and the extinction coefficients calculated from their amino acid sequences.

### Antibacterial Assays

Cyclotides (cycloviolacin O2, O3, and O19) at concentrations of 0.78–100 μM were tested for activity against three bacterial plant pathogens: *Pseudomonas syringae* pv. *syringae* strain Pss-762 (Taraszkiewicz et al., 2012), *Dickeya dadantii* strain 3937 (Glasner et al., 2011) and *Pectobacterium atrosepticum* SCRI 1043 (Bell et al., 2004). Experiments were conducted according to the CLSI guidelines (Clinical and Laboratory Standards Institute [CLSI], 2012) using standard CA-MHB medium (BBL^TM^ Mueller-Hinton II Broth cation-adjusted, Becton Dickinson) or M9 minimal medium (per L: 6 g Na_2_HPO_4_, 3g KH_2_PO_4_, 0.5 g NaCl, 1 g NH_4_Cl) supplemented with glucose to a final concentration of 0.4%. Bacterial colonies from 24 h old cultures on TSA (trypticase soy agar, BioMerieux) plates were used to inoculate CA-MHB or M9 minimal medium and set up liquid cultures. Next, late log phase bacterial cultures were diluted to 0.5 McFarland standard, as determined by densimetry in the relevant medium (DensiMeter II, EMO, Brno). The final inoculum concentration used in the assays was 1.5 × 10^6^ Colony Forming Units (CFU) ml^-1^. The cultures were then incubated with a cyclotide at a specific concentration for 24 h at 28°C in the case of *P. syringae* pv. *syringae*, 37°C in the case of *D. dadantii*, and 25°C in the case of *P. atrosepticum*. The minimal inhibitory concentration (MIC) of each cyclotide was determined as the lowest concentration required to inhibit bacterial growth, i.e., to reduce the culture’s OD_600_ to less than 0.1. OD_600_ values were measured with a microplate reader (EnVision^TM^ Multilabel Plate Reader, Perkin Elmer). Every tested cyclotide concentration was assayed in triplicate, and the experiment as a whole was repeated three times.

### Antifungal Assays

Four cycloviolacin cyclotides (cyO2, cyO3, cyO13, and cyO19) were tested against seven fungal plant pathogens: *Fusarium oxysporum* Schlecht.: (Levenfors et al., 2004), *Fusarium graminearum* Schwabe, *Fusarium culmorum* Sm. Sacc.; *Mycosphaerella fragariae* (Tulasne) Lindau, *Botrytis cinerea* Pers.; Fr., *Colletotrichum utrechtense* Damm. and *Alternaria alternata* (Fr.) Keissl. Appropriate amounts of stock peptide solutions were freeze dried and resuspended in Milli-Q water or growth medium. Standard microdilution antifungal assays as described by Broekaert et al. (1990) were performed using 24 g l^-1^ potato dextrose broth (PDB, Sigma-Aldrich St. Louis, MO, United States) or 12 g l^-1^ PDB in flat-bottomed 96-well Nunc A/S plates (Thermo Scientific). *F. oxysporum* was kindly supplied by Dr. Jolanta Levenfors (Levenfors et al., 2004) in the form of a frozen spore suspension (macroconidia). *B. cinerea, F. graminearum, F. culmorum* and *M. fragariae* were obtained from the Institute of Plant Protection – National Research Institute, Poznań, Poland (accession no. 2235, 2172, 2169, 1089 respectively). *C. utrechtense* and *A. alternata* were isolated from plants of the violet species *V. uliginosa* and *V. odorata* respectively. The *V. uliginosa* plants used for this purpose exhibited no signs of disease, but the *V. odorata* plant had a small (3 mm in diameter) necrosis spot on one leaf. Fungi were isolated from leaf fragments cultured on potato dextrose agar (PDA, Sigma-Aldrich) after surface sterilization by rinsing with 70% ethanol for 1 min and commercial bleach in distilled water (1:3) for 7 min, followed by washing 3 times with distilled water. The growing fungi were subcultured on PDA (Sigma-Aldrich), then liquid cultures were obtained by inoculation to PDB. The two isolated fungal species were identified by The Westerdijk Fungal Biodiversity Institute (Utrecht, Netherlands).

Fungal cultures were maintained in darkness at room temperature (RT) and liquid cultures on a rotary shaker. All manipulations were performed under aseptic conditions to avoid contamination. Fresh *F. oxysporum* and *M. fragariae* spores (microconidia) were obtained from 5 days old cultures in PDB (24 g l^-1^) filtered through cheese cloth, centrifuged, resuspended in fresh growth media and inoculums densities were measured with hematocytometer. *B. cinerea* spores were harvested from 7 days old cultures on malt agar (30 g l^-1^, Sigma-Aldrich) whereas *F. graminearum, F. culmorum* from 2 week old cultures on SNP (synthetic nutrient poor) media: glucose 0.2 g l^-1^, sucrose 0.2 g l^-1^, potassium dihydrogen phosphate 1 g l^-1^, potassium nitrate 1 g l^-1^, magnesium sulfate anhydrous 0.25 g l^-1^, potassium chloride 0.5 g l^-1^, agar 14 g l^-1^ (all ingredients supplied by Sigma-Aldrich). To isolate the spores, agar plates with fungal cultures were rinsed with deionized, sterile water and the supernatant was filtered through cheese cloth. The spore densities were measured with a hematocytometer, suspensions were centrifuged, resuspended in 20% glycerol and stored at -80°C. *A. alternata* mycelia fragments suspension was used for the assays. Plaques of mycelia from 5 days old culture in PDB (24 g l^-1^), 1–2 cm in diameter, were macerated with a glass rod in glass vial and then filtered through three layers of cheese cloth. The filtrate was centrifuged, resuspended in fresh medium and the mycelia fragments (42 μm long, mean of 100 measurements, *SD* = 20) density was measured with a hematocytometer.

Spore and mycelia fragments suspensions for assays were prepared from stocks (10^6^–10^7^ spores ml^-1^, stored in 20% glycerol at -80°C) or from fresh cultures; in both cases, the inoculum densities were adjusted using a hematocytometer. Prior to the assays, the suspensions were centrifuged and re-suspended in fresh 24 g l^-1^ PDB to obtain final concentrations of 2000, 1000, or 500 spores per well or 2000 and 4000 mycelia fragments per well in the assays (total volume per well = 100 μl).

Minimal inhibitory concentrations values were determined by measuring the difference between the optical density (OD, absorbance at 600 nm) at the start of the incubation with a peptide (0 h) and that after 48 h (in the case of *F. oxysporum, F. graminearum, F. culmorum, B. cinerea*, and *M. fragariae*) or 72 h (in the cases of *C. utrechtense* and *A. alternata*) using a Varioskan Flash Multimode Reader (Thermo Scientific). The difference lower than 0.1 indicated growth inhibition. Each antifungal assay consisted of three replicates and the whole experiments were performed at least twice on different occasions.

### Cyclotide Stability Assay

Freshly harvested *F. oxysporum* spores were grown in microplates (3000 spores in 150 μl of 24 g l^-1^ PDB per well) on a rotary shaker. After 5 days of incubation, 5 μg of cyO2 in 10 μl of Milli-Q water was added to each well. Samples of the medium and mycelia were collected in Eppendorf tubes immediately after adding cyO2 (time 0) and after incubation with the cyclotide for 2, 4, 8h, 1, 2, and 3 days. The samples were freeze-dried, crushed with a pestle, and extracted with 200 μl of buffer B for 4 h on the rotary shaker. After centrifugation, the supernatant was collected, freeze-dried and reconstituted in buffer A for analysis by HPLC-UV-ESI-MS. HPLC was performed over 18 min using an LC-20 UFLC system (Shimadzu, Japan) with photodiode array detector (PDA) and a Waters (Milford, MA) XTerra MS C18 HPLC column (2.1 × 50 mm, with a particle size of 2.5 μm) at a flow rate of 0.5 ml min^-1^ with a linear gradient from 5% to 60% ACN and 0.1% formic acid in water. ESI-MS detection was used to identify the cyO2 peak and to detect modifications of the peptide that may have been introduced by the fungus. For each incubation time, the peaks in the 280 nm UV chromatogram corresponding to cyO2 were integrated, and the mean area under the curve (AUC) for each incubation time was computed from independent triplicates. The percentage of intact peptide at each incubation time was then computed as the difference between the mean AUC for that time point and that at time 0, divided by the AUC at time 0. The cyclotide was considered stable after incubation for a given length of time if the percentage of intact peptide for that incubation time was above 80%.

### Liposome Leakage Assays

Liposomes were prepared and their permeabilization was assayed as described previously (Strömstedt et al., 2016). To obtain liposomes resembling fungal membranes, the liposomes were prepared from a mixture of a *Saccharomyces cerevisiae* polar lipid extract (Avanti Polar Lipids, Alabaster, AL, United States) and ergosterol (Sigma-Aldrich) in a 7:3 molar ratio. Briefly, dried lipid films were formed on round-bottom flask walls and re-suspended at 55°C with Tris buffer containing 100 mM 5(6)-carboxyfluorescein. The suspensions were then subjected to repeated extrusion through a 100 nm polycarbonate membrane to break up multilamellar structures and reduce polydispersity. Un-trapped carboxyfluorescein was removed by gel filtration. Membrane permeability was measured by monitoring the efflux of carbofluorescein from liposomes, which reduces its self-quenching and thus increases its fluorescence. Two-fold serial dilutions in Tris buffer were prepared in separate 96-well plates for each cyclotide to be tested, and the liposome suspension was added to each well of each serial dilution plate. The peptide solutions’ effects on carbofluorescein efflux were then monitored over 45 min, by which time the leakage had largely subsided. The reported results are means and standard deviations based on triplicate experiments, and are expressed as percentages of the total leakage induced by Triton X-100. The reported EC_50_-values are calculated from a sigmoidal dose-response curve in which the leakage percentage is plotted as a function of the peptide concentration (log10).

### Immunogold and Transmission Electron Microscopy of *F. oxysporum* Spores Treated With cyO2

Immunogold labeling for TEM requires relatively high inoculum densities, necessitating the use of high cyclotide concentrations to reach the MIC. A preliminary evaluation indicated that the optimal *F. oxysporum* spore concentration in terms of effective immunogold labeling and the peptide concentration needed to exceed the MIC was 5 × 10^5^ in 1 ml. The corresponding MIC for cyO2 in PDB (12 g/ml) was 80 μM. Consequently, a peptide concentration of 100 μM was used in all subsequent immunogold labeling and TEM experiments.

*F. oxysporum* spores were obtained from fresh cultures as described above and suspended in 1 ml of fresh PDB (12 g/ml) with cyO2 in an Eppendorf tube. After 6 h, the spores were pelleted at 6000 RPM for 5 min, re-suspended in a fixation buffer consisting of 4% formaldehyde (freshly prepared from paraformaldehyde) and 0.25% glutaraldehyde in PBS, and incubated overnight at 4°C. After fixation, spores were washed for 3 × 10 min in PBS, dehydrated in a graded ethanol series (10–100%), and embedded in LR-white resin (Sigma-Aldrich). Ultrathin sections were cut on a Leica EM UC7 ultramicrotome equipped with a diamond knife (DIATOME ultra 45°, Diatome Ltd, Switzerland), collected on gold grids, and blocked with 3% BSA (bovine serum albumin) in PBS buffer for 1 h at RT. The sections were then incubated with the primary antibody (1:1000 dilution) for 24 h at 4°C, rinsed four times in PBS, and incubated with the secondary antibody conjugated with 10 nm gold particles (Anti-Rabbit IgG [whole molecule] – Gold antibody produced in goat, Sigma-Aldrich) at a dilution of 1:50 for 4 h at RT, as recommended by the manufacturer. After washing with distilled water, the ultrathin sections were post-stained with 2% (w/v) uranyl acetate (Sigma-Aldrich) and Reynold’s lead citrate (Sigma-Aldrich) (Vaughn, 2013). Samples were examined using an FEI Tecnai G2 Spirit TWIN/BioTWIN transmission electron microscope (FEI Company, Hillsboro, OR, United States). The experiments were done in triplicates and the conclusions were drawn from at least 3 stained grids per treatment and hundreds of observed spores.

### Immunostaining and Fluorescence Microscopy

The primary anti-cycloviolacin cyclotides antibodies obtained in a previous study and established protocols for immunostaining were applied to *V. odorata* flowers and whole seeds (Slazak et al., 2016).

### Matrix-Assisted Laser Desorption Ionization Mass Spectrometry Imaging (MALDI-MSI)

Fragments of the petiole, roots, and leaves (including fragments incorporating the main vein and fragments of tissue from the regions between veins) were subjected to one of three different pretreatments: (1) embedding in ice; (2) embedding in gelatin (100 mg/mL in Mili-Q water) and freezing on dry ice; or (3) infiltrating with PBS buffer under vacuum for 15–20 min to eliminate air bubbles from the tissues, followed by embedding in gelatin and snap-freezing in liquid nitrogen. Tissues were sectioned into 16–20 μm thick slides using a cryostat microtome (Leica CM1900 UV, Leica Microsystems, Welzlar, Germany) and thaw-mounted onto conductive indium tin oxide (ITO) glass slides (Bruker Daltonics, Bremen, Germany). Sections were dried gently under a flow of nitrogen and then placed in a desiccator in RT for 15 min. MALDI matrix solution (2,5-dihydroxybenzoic acid, 35 mg/mL in 50% acetonitrile, 0.2% TFA) was sprayed over the samples using an automatic matrix sprayer (TM-Sprayer; HTX Technologies, Carrboro, NC, United States) at a flow rate of 70 μl/min with a stage speed of 1100 mm/min, track spacing of 2 mm (crisscross pattern), nitrogen pressure of 6 psi, and a spray nozzle temperature set to 98°C for 8 passes. Samples were scanned using a flatbed scanner (Epson Perfection V500) to obtain optical images (9600 dpi). MALDI-MSI analyses were done using a rapiflex MALDI TOF/TOF (Bruker Daltonics) in positive ion reflectron mode using a Smartbeam 10 kHz laser. The MS acquisition parameters were as recommended by the manufacturer, adjusted for optimal performance. Data collection was performed at a spatial resolution of 10 μm, with 300 laser shots at each fixed raster position. Tissue sections were analyzed in a random order to prevent bias due to factors such as matrix degradation or variation in mass spectrometer sensitivity. To produce an image of a particular cyclotide, its average singly charged signal ± 2.5 Da was used to cover the different ions from the peptide’s isotopic pattern. The *m/z* values for the singly charged cyclotides are: cyO2 = 3140.69; cyO3 = 3153.39; cyO14 = 3178.34; cyO19 = 3227.42; kB1 = 2892.28; varv A/kalata S = 2878.25. MSI data were analyzed and normalized against total ion counts (TIC) using FlexImaging, version 4.0 (Bruker Daltonics).

### Extraction of Cyclotides From Dissected Tissues and Liquid Chromatography-Mass Spectrometry (LC-MS) Analysis

**FIGURE 1:**
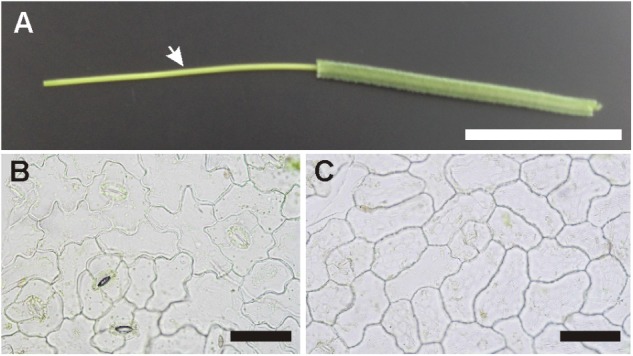
Isolation of different *V. odorata* tissues. **(A)** Petiole fragment with the vascular bundle (marked with an arrowhead) uncovered by removing the surrounding tissues. **(B)** Isolated fragments of lower (abaxial) and **(C)** upper (adaxial) leaf epidermis. Bar = **(A)** 1 cm; **(B,C)** 100 μm.

Cyclotide expression was compared in isolated *V. odorata* petiole vascular bundles, adaxial (upper) and abaxial (lower) leaf epidermis samples, and the corresponding whole organs (i.e., petioles and leaves). In addition, cyclotide expression patterns were characterized in *V. odorata* embryos and endosperm. Vascular bundles were isolated by bending the petiole until it broke, allowing the sheathing tissues to be removed by pulling the ends of a broken fragment (**Figure 1A**). Three replicate samples were analyzed, each of which was pulled from 3-4 petiole fragments from different plants. To separate the upper and lower epidermis, the leaf blades were cut with a razor blade at a very sharp angle to obtain a single layer of cells at the cut edge. By grabbing the edge with a forceps and pulling vigorously, small fragments of epidermis were ripped off (**Figures 1B,C**). Material from different *V. odorata* specimens and positions on the leaf was pooled into three replicates to average out all variation in cyclotide expression other than that between different tissues. *V. odorata* seeds were dissected to separate embryos and endosperm.

The *V. odorata* tissues and organs were freeze-dried immediately after preparation, extracted, and analyzed by LC-MS using established procedures (Slazak et al., 2015).

## Results

### Cycloviolacin Cyclotides Have Potent Activity Against Plant Pathogenic Fungi

The antimicrobial activity of cyO2, cyO3, cyO13, and cyO19 was determined to test the hypothesis that cyclotides are a part of a plant defense mechanism in *V. odorata*. To this end, cyclotides were tested against seven fungal plant pathogens: *F. oxysporum, F. graminearum, F. culmorum, Mycosphaerella fragariae, Botrytis cinerea, Colletotrichum utrechtense*, and *Alternaria alternata*. The first five of these species are model plant pathogens. The latter two were isolated from *V. uliginosa* and *V. odorata*, to evaluate the activity of cyclotides against pathogens present in the plants’ environment.

All of the tested cycloviolacin cyclotides exhibited low micromolar activity against every tested fungus (**Table 1**). The most potent cyclotide was cyO3 (MICs ranging from 0.8 to 12.5 μM), and the least active was cyO13 (MICs ranging from 3 to 25 μM). However, in all cases but one, the differences between peptides were within one dilution factor (x2). The exception was that substantially larger differences were observed between *A. alternata* and *F. oxysporum*: the former was the most susceptible fungus to the tested cyclotides, with MICs ranging from 0.8 to 3 μM, while the latter was the least sensitive, with MICs ranging from 6.25 to 12.5 μM. The cyclotides’ selectivity for *A. alternata* over *F. oxysporum* thus ranged from x4 to x8. Note that the most sensitive fungus was the *V. odorata* pathogen, and cyclotides were isolated from this plant.

**Table 1:** The MICs of four cycloviolacin cyclotides (cyO2, cyO3, cyO13, cyO19), varv A (kalata S) and kB1 against seven fungal plant pathogens.

	PDB	MIC (μM)					
		cyO2	cyO3	cyO13	cyO19	varv A	kB1
*F. oxysporum*	24 g l^-1^	12.5	12.5	25	25	nd	nd
	12 g l^-1^	6.25	6.25	12.5	12.5	nd	nd
*B. cinerea*		1.5	1.5	3	1.5	80	80
*F. graminearum*		6.25	nd	nd	12.5	40	>80
*F. culmorum*		1.5	nd	nd	1.5	40	>80
*M. fragariae*		>25	nd	nd	>25	>80	>80
*C. utrechtense*		6.25	6.25	6.25	6.25	nd	nd
*A. alternata*^m^		1.5	0.8	3	1.5	nd	nd

*MIC, minimal inhibitory concentration; PDB, potato dextrose broth; nd, no data; m, mycelia fragments, 4000 per well*.

The most abundant cyclotide in *V. odorata*, cyO2, had twice the activity of cyO19 against *F. oxysporum* (MIC = 6.25 μM and 12.5 μM, respectively) but had similar MICs against *C. utrechtense* and *A. alternata* (6.25 μM and 1.5 μM, respectively). It also exhibited high activity against *B. cinerea* (MIC between 1.5 and 3 μM).

The two tested kalata type cyclotides – varv A (kalata S) and kB1 – exhibited very low activity against the tested fungi when compared with cycloviolacins, with MICs equal to or greater than 80 μM (**Table 1**).

The peptides’ activity varied with the growth medium composition – the MIC values in 24 g l^-1^ PDB were twice as high as those obtained in 12 g l^-1^ media (**Table 1**). The inoculum density had less influence on the measured activity: the MIC values observed in experiments with 500 spores or mycelial fragments per well were identical to those using 2000 spores or fragments per well.

### Cyclotides Disrupt Fungal Membranes and Flow Into Spore Cytoplasm

**FIGURE 2:**
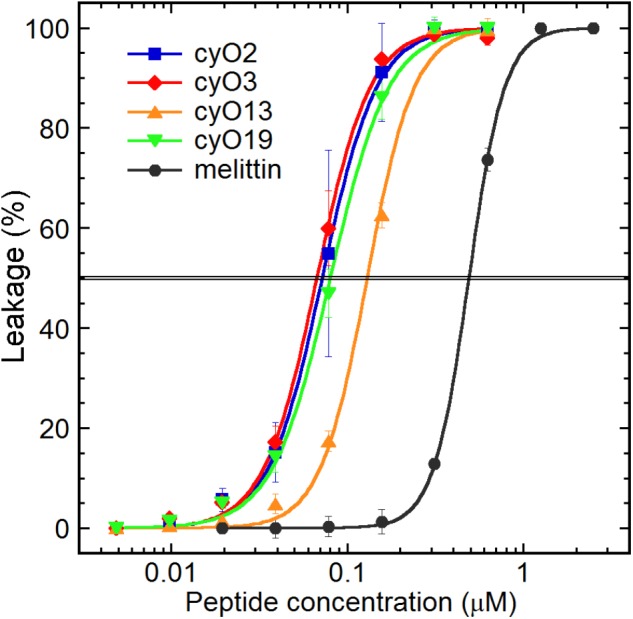
Permeabilization of a fungal cell membrane model by cycloviolacin cyclotides from *V. odorata*: cyO2, cyO3, cyO13, cyO19 (EC_50_ = 72 nM; 67 nM; 130 nM; 80 nM respectively). All the tested cyclotides exhibited similar effectiveness at disrupting fungal lipid membranes, and were approximately 4–7 times more active than the bee venom antimicrobial peptide melittin (EC_50_ = 490 nM).

The ability of cyclotides to disrupt model fungal membranes was investigated to clarify their mechanism of action. All four cycloviolacins showed strong membrane permeabilization activity in the liposome assay (**Figure 2**), and the order of their potency mirrored that of their antifungal activity: cyO2, cyO3 and cyO19 are equipotent, with EC_50_ values of 67–80 nM, whereas cyO13 is less potent, having an EC_50_ of 130 nM. Notably, the cyclotides permeabilized the model fungal membranes at concentrations approximately 4–7 times lower than were required to achieve comparable permeabilization with the control peptide melittin.

**FIGURE 3:**
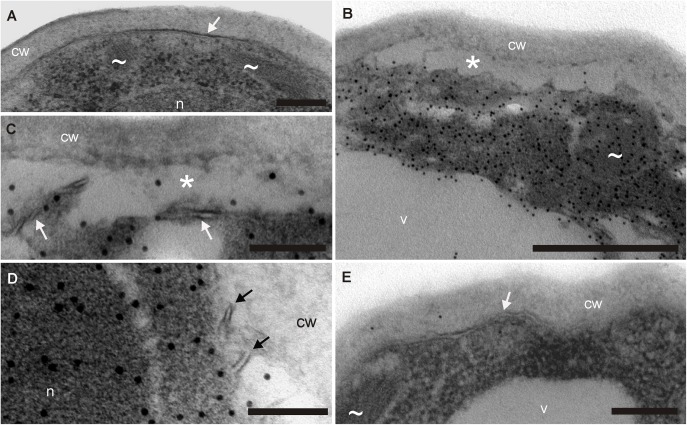
Demonstration of cyclotides’ antifungal mode of action at the ultrastructure level –TEM images of *F. oxysporum* spores incubated with cyO2 and stained with immunogold. **(A)** Negative control – spores subjected to the complete immunogold procedure but not treated with the cyclotide. No gold particles are visible, demonstrating the antibodies’ specificity. The spores had normal cell walls (cw), cell membranes (arrowheads), mitochondria (∼) and nuclei (n). **(B–D)** Spore cells damaged by treatment with cyO2 – fragmented cell membranes (arrows) and plasmolysis (^∗^). The cyclotide was found in all the cell structures (indicated by the presence of gold particles): the cytoplasm, mitochondria and nucleus, although only very small amounts were observed in the vacuole (v). **(E)** A single undamaged cell that did not bind cyO2 among the treated spores. The TEM images are representative of at least 3 stained grids per treatment and hundreds of observed spores. Bar = **(C,D)** 100 nm; **(A,E)** 200 nm; **(B)** 500 nm.

Immunogold labeling and TEM confirmed that cyclotides induce membrane disruption in living cells. **Figure 3** shows the damage suffered by *F. oxysporum* spore cells after cyO2 treatment. All negative controls (no treatment, omitting primary or secondary antibody) were free of gold particles, demonstrating the specificity of the anti-cyO2 antibody (**Figure 3A**). The cyO2-treated spores exhibited disrupted plasmolemmas and clear signs of osmotic stress, and plasmolysis compared to control spores grown in the absence of the peptide (**Figures 3A–D**). The peptide was observed in every structure within the fungal cells, but was only present in small quantities in the vacuoles. Among hundreds of treated cells, we observed only one that was seemingly unaffected and had no internalized cyclotide molecules (**Figure 3E**).

### Cyclotides Are Stable in the Presence of Fungal Plant Pathogens

**FIGURE 4:**
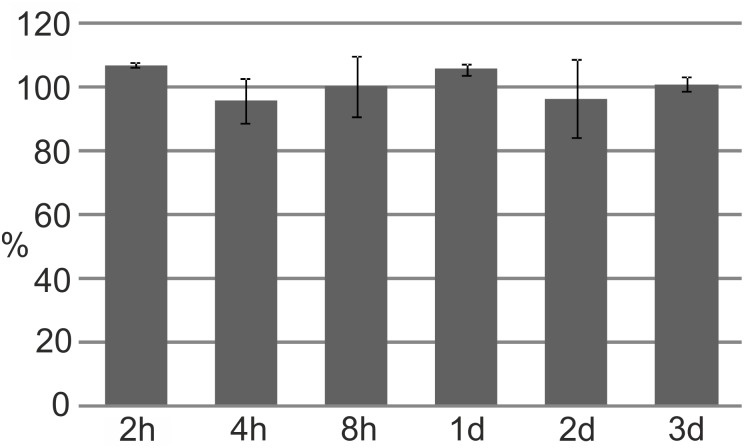
Cyclotides’ resistance to degradation by the fungal plant pathogen *F. oxysporum*. The proportion of non-degraded cyO2 (measured by integrating the AUC for the peptide peak at 280 nm) relative to that at time 0 after 2 h (h) to 3 days (d) of incubation with the fungus. All results are mean values of three replicate experiments, bars indicate standard deviations.

The stability of cyclotides in the presence of *F. oxysporum* was tested by monitoring the levels of the peptides after different incubation times. The fungus proved incapable of breaking down the cyclotide structure; even after 3 days of co-culture, almost 100% of the initially added amount of cyO2 could be recovered, with an unaltered molecular mass (**Figure 4**).

### Cyclotides From *V. odorata* Show Moderate Activity Against Plant Pathogenic Bacteria

Having established the effect of cyclotides on fungal pathogens, we explored their activity against bacterial plant pathogens to better understand their possible roles in host defense. Experiments were performed on *Pseudomonas syringae* pv. *syringae, Dickeya dadantii*, and *Pectobacterium atrosepticum*, in CA-MHB and M9 minimal media. In CA-MHB media, the MIC values for all three cyclotides against the tested bacterial strains were greater than the highest tested cyclotide concentration (100 μM). However, all of the peptides exhibited moderate antibacterial activity in experiments using minimal medium. All three cyclotides had similar MICs against all three tested bacterial strains – 25 μM for *P. syringae*, 75 μM for *P. carotovorum* and 100 μM for *D. dadantii* (**Table 2**).

**Table 2:** The MICs of cycloviolacin cyclotides (cyO2, cyO3, cyO19) toward three bacterial plant pathogens.

	MIC (μM)		
	cyO2	cyO3	cyO19
*P. syringae pv. syringae*	25	25	25
*D. dadantii*	75	75	75
*P. atrosepticum*	100	100	100

*MIC, minimal inhibitory concentration*.

### Linking Biological Activities With Distribution – Cyclotides in Different Plant Tissues

**FIGURE 5:**
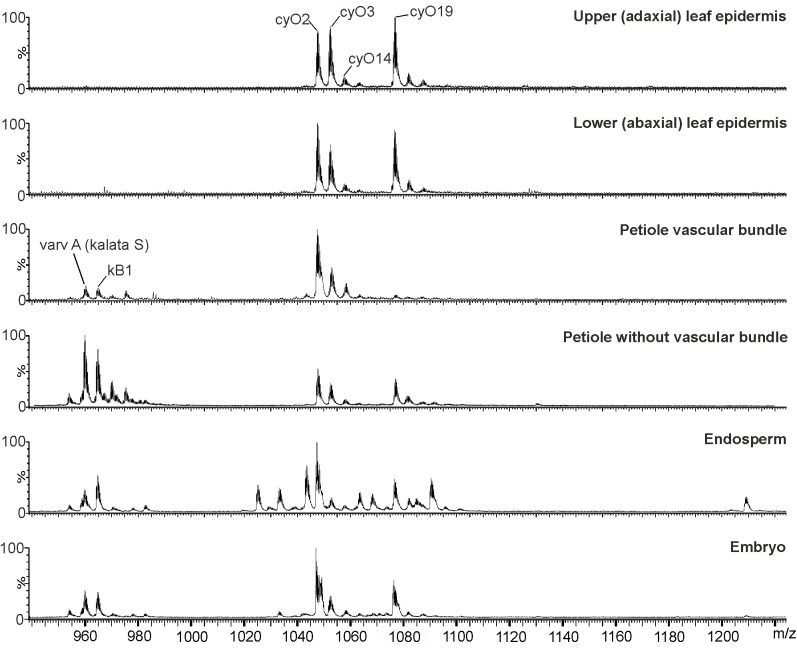
MS profiles of cyclotide extracts from different *V. odorata* isolated tissues and parts of the seed: adaxial and abaxial leaf epidermis, petiole vascular bundles, endosperm and embryo. Cycloviolacin cyclotides (cyO2, cyO3, cyO14, and cyO19) dominate in the leaf epidermis and vascular bundles. Quantities of kalata B1 and S found in the vascular bundle were much smaller than those in the petiole with the vascular bundle removed. Both kalata and cycloviolacin cyclotides were present in the dissected seed parts. The endosperm exhibited the highest cyclotide diversity. The figure shows results for triply charged (3+) ions.

**FIGURE 6:**
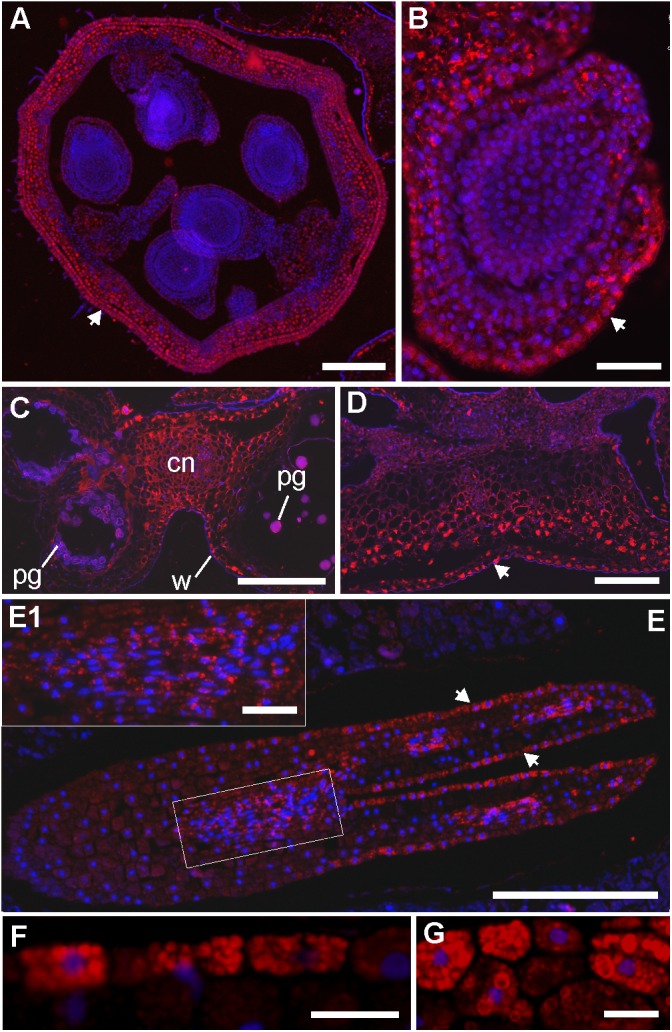
Immunolocalization of cycloviolacin cyclotides (cyO2, cyO3, cyO13, cyO19) in the ovary, ovule, anther, mature embryo and endosperm of *V. odorata*. DAPI and Daylight 549 channels are merged, and the locations of cyclotides and nuclei are revealed by red and blue fluorescence, respectively. **(A)** Cross section of the ovary and **(B)** longitudinal section of the ovule; large amounts of cyclotides were found in the ovary wall and integuments (marked with arrowheads). **(C)** Cross section of the anther with visible pollen grains (pg) and cyclotides in the connective (cn) and the pollen sac walls (w). **(D)** Cross section of the appendix shielding the anther in the flower, with large amounts of cyclotides, especially in the epidermis (marked with an arrowhead). **(E)** Longitudinal section of the mature embryo – cyclotides are present in all the tissues, with higher amounts in the protodermis (arrowheads) and procambium **(E1)**. **(F)** Close-up on the embryo protoderm and **(G)** endosperm cells with small vacuoles/protein bodies filled with cyclotides. Bar = **(A,C,D,E)** 250 μm; **(B,E1)** 50 μm; **(F,G)** 20 μm.

LC-MS and immunohistochemistry experiments were then used to explore the link between the cyclotides’ antimicrobial activities and their locations in *V. odorata* flowers and seeds. We first showed that cyO2, cyO3 and cyO19 are present in both embryos and the endosperm during the earliest stages of plant development by using LC-MS to analyze extracts from dissected seeds (**Figure 5**). The greatest variation in terms of cyclotide sequences was found in the endosperm. We also examined the distribution of cycloviolacin cyclotides in the ovary, anther, embryo, and endosperm applying immunohistochemistry (**Figure 6**). The ovary walls (**Figure 6A**) and the outer integuments of the ovule (**Figure 6B**) both exhibited substantial cycloviolacin accumulation. Large amounts of cyclotides were also detected in the anther connective and pollen sac walls (**Figure 6C**), the appendix shielding the anther (**Figure 6D**), the embryo protodermis, and the procambium (**Figures 6E,E1**). In the protodermis and endosperm cells, cyclotides were found in multiple small vacuoles/protein bodies (**Figures 6F,G**).

**FIGURE 7:**
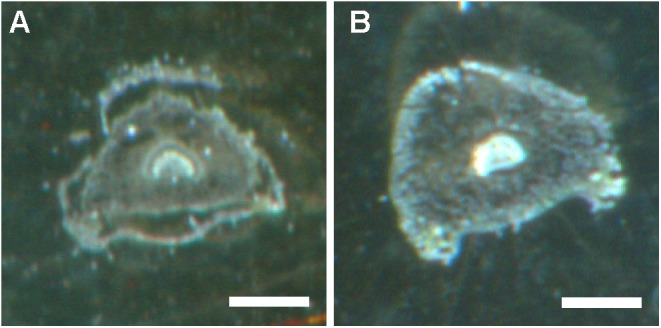
Preservation of tissue structure in *V. odorata* petiole cross sections obtained using different pretreatments, embedment techniques, and freezing temperatures. **(A)** Embedment in gelatin and freezing on dry ice. **(B)** Infiltration with PBS buffer under vacuum, embedment in gelatin, and snap freezing in liquid nitrogen. Optical images were taken before matrix application. Bar = 500 μm.

**FIGURE 8:**
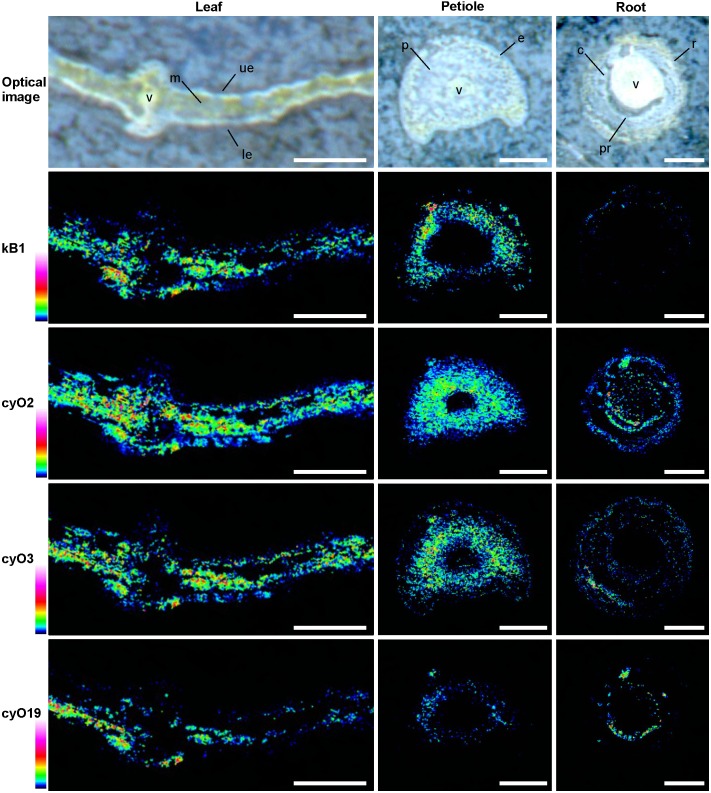
Distribution of the cyclotides kB1, cyO2, cyO3, and cyO19 in *V. odorata* leaf, petiole and root cross sections, depicted by MALDI-MSI. Warmer colors indicate higher abundance. kB1 was detected in the leaf mesophyll (m), close to the adaxial (ue) and abaxial (le) leaf epidermis, the outer layers of the petiole parenchyma (p), and the epidermis (e), but not in the roots. Cycloviolacin cyclotides were localized to the leaf mesophyll and epidermis, in and close to the vascular bundle (v), and in various tissues of the root - the pericycle (pr), cortex (c) and rhizodermis (r). Optical images were acquired after matrix application. Bar = 500 μm.

**FIGURE 9:**
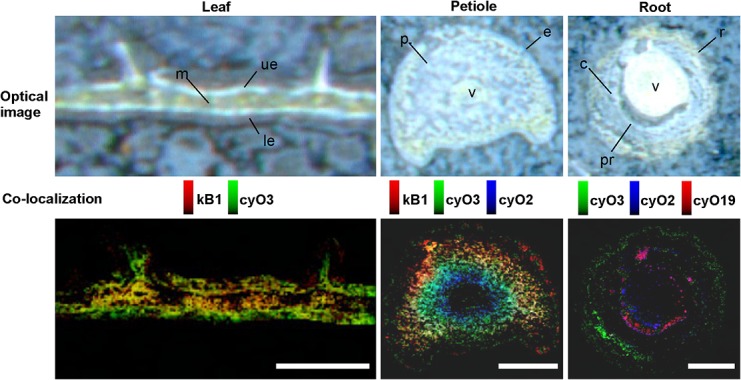
Distribution and co-localization of different cyclotides in *V. odorata* leaf, petiole and root cross sections, determined by MALDI-MSI. The distinct spatial distributions of kB1, cyO2, cyO3, and cyO19 are clearly visible in the co-localization images – cycloviolacins occur in the adaxial (ue) and abaxial (le) leaf epidermis, close to and in the petiole vascular bundles (v) and root cortex (c), rhizodermis (r), and pericycle (pr). Optical images were acquired after matrix application. kB1 found in leaf mesophyll (m) and petiole parenchyma (p). Bar = 500 μm.

We subsequently used high spatial resolution MALDI-MSI and LC-MS analyses of extracts from dissected tissues (the abaxial and adaxial epidermis and vascular bundle, **Figure 1A**) to determine the distributions of both cyclotide types found in mature plants: kalata (notably kB1) and cycloviolacin (notably cyO2, cyO3, cyO19). We initially evaluated different pretreatment, embedding, and sectioning techniques for MALDI-MSI. Embedment in ice was not applicable — the tissues broke down completely as the sections were thaw-mounted on conductive slides. The best tissue integrity was achieved when air was rigorously excluded from the sections by infiltration with PBS under vacuum before embedment in gelatin and then snap-freezing in liquid nitrogen (**Figure 7**). However, even with this optimized technique, some tissue shrinkage was observed that resulted in a slight mismatch between the MALDI-MSI analyses and optical images of leaf cross-sections (**Figures 8**, **9**).

**FIGURE 10:**
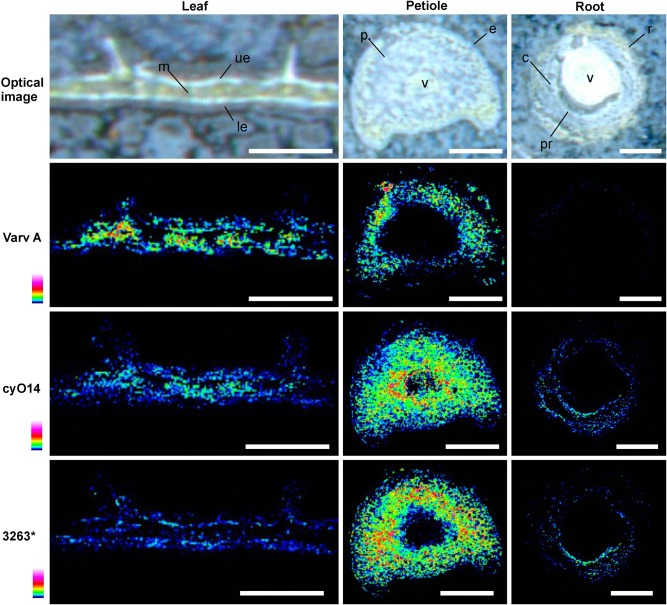
Distribution of different cyclotides in *V. odorata* leaf, petiole and root cross sections as determined by MALDI-MSI. The locations of cyclotides were determined based on the distribution of the corresponding high intensity peaks in the average MALDI mass spectra, and were as follows: varv A - in the outer layers of the petiole parenchyma (p); cyO14 - leaf mesophyll (m), petiole parenchyma (p), and vascular bundles (v); 3263 - petiole parenchyma (p) and leaf epidermis (ue, le). varv A was absent from the roots. Warmer colors indicate higher abundance. Optical images were acquired after matrix application. Bar = 500 μm.

MALDI-MSI and LC-MS analyses of extracts showed that cyo2, cyO3, cyO19 dominated in the leaf epidermises, vascular bundles, and roots (**Figures 8**, **9**). These cyclotides were also found in the ground tissues — the petiole collenchyma and root pericycle — close to vascular bundles. kB1 was associated with the petiole parenchyma and leaf mesophyll, and was almost absent from the root. In the petiole, this peptide is localized in the outside layers of the parenchyma, far from the vascular bundle (**Figure 8**). This distribution pattern of kB1 is in agreement with LC-MS analysis of extracts from dissected tissues. Substantially higher kB1 levels were observed in the tissues surrounding the vascular bundle (**Figure 10**). The distinct distributions of different cyclotides are clearly visible in the co-localization MS images (**Figure 9**). The average mass spectra also indicated the presence of additional cyclotides. varv A (kalata S) exhibited a similar distribution to kB1, being present in the petiole parenchyma and at low levels in the root. Another two peptides – cyO14 and 3263 (an unknown cyclotide that is referred to by its monoisotopic mass) – were found in the vascular bundles and petiole parenchyma, respectively (**Figure 10**).

## Discussion

This work presents a thorough analysis of *V. odorata* defense mechanisms based on cyclotides. The results support the notion of cycloviolacin cyclotides playing a role in defense against plant pathogens and depicts the mode of their antifungal action. Additionally, we connect the distributions of different cyclotides, cycloviolacins and kalata B1 with specific activities, to their biological functions - antimicrobial and anti pest defense.

We first showed that cyclotides produced by *V. odorata*—namely cycloviolacins O2, O3, O13, and O19—have potent activity against fungal plant pathogens. Many cysteine-rich knotted plant peptides other than cyclotides (e.g., defensins) play roles in antifungal host defense (van der weerden et al., 2013; Goyal and Mattoo, 2014). However, so far, only one cyclotide (cyO8) has been tested recently for activity against *F. gramineum* (Parsley et al., 2018). The assayed peptides were generally more potent against the two fungal pathogens (*C. utrechtense* and *A. alternata*) isolated from violets and *B. cinerea* than against *F. oxysporum*. The comparatively low MIC values against the latter species may indicate specialization of individual cyclotides and their co-evolution with pathogens present in the host plant’s environment. The different MIC values for *F. oxysporum* spores and *A. alternata* mycelia may be explained by their distinctive biology and morphology. This seems consistent with cyclotides having a role in plant host defense, because fungi spread and invade plant tissues as mycelia (Knogge, 1996), whereas spores are harmless until germination. *A. alternata* was isolated from a spot with visible signs of disease but the biology of the interaction of *V. odorata* with pathogens is yet to be investigated. It may be that the infection was somehow opportunistic, in preexisting necrosis or in other way weakened tissues. The present study indicate that the defense mechanism based on cyclotides can contribute greatly to confinement of the infection to this one spot—prevention of the systemic spread of the pathogen. Low activity of varv A (kalata S) and kB1 against the tested pathogens support the hypothesis that different types of peptides specialize to play particular biological roles. kB1 was found to be active against insect larvae (Jennings et al., 2001). The composition of the assay growth medium affected the cyclotides’ activity: different results were obtained when the growth medium was changed from 12 g l^-1^ PDB to 24 g l^-1^ PDB. However, variation in the concentration of the PDB medium (which consists mainly of carbohydrates) had a much less pronounced effect than replacing PDB with more complex media. Earlier studies on human pathogens showed that the antimicrobial activity of cyclotides diminishes in rich media such as tryptic soy broth (TSB) supplemented with high salt concentrations (Tam et al., 1999; Pränting et al., 2010).

The cyclotides’ antifungal activity seems to be connected to membrane disruption. Three different cycloviolacins (cyO2, cyO3, and cyO19) have been shown to permeabilize model bacterial membranes to similar degrees, achieving EC_50_ values of 76–110 nM against Gram-negative *Escherichia coli* lipid liposomes (Strömstedt et al., 2017). In this work, the same peptides showed similar levels of activity against model fungal membranes. Notably, their effects were 4–7 times stronger than that of the control peptide melittin, which analogs are some of the most active known peptides in membrane disruption assays (Asthana et al., 2004).

Cyclotides are known to selectively target and disrupt membranes containing phosphatidylethanolamine (PE) (Burman et al., 2011; Strömstedt et al., 2017); their proclivity for membrane-binding increases with the membrane’s content of PE as a proportion of its total phospholipids (Kamimori et al., 2005; Henriques et al., 2011). This influences activity and selectivity because there are substantial differences in membrane lipid composition between organisms. However, the *S. cerevisiae* lipid extract used to prepare the model fungal membranes examined in this work has a PE-lipid content of only 12%, whereas the PE-lipid content of comparable *E. coli* extracts is 67%. In both these model membranes, anionic phospholipids account for approximately one third of the total lipids. This suggests that other phospholipid- or sterol-dependent factors make fungal membranes particularly susceptible to permeabilization by cyclotides. Regardless, the liposome permeabilization data corroborate the potent antifungal properties of these cyclotides and implicate membrane disruption as their antifungal mechanism of action.

Transmission electron microscopy imaging of *F. oxysporum* spores treated with cyO2 supports the hypothesis that they act against fungi by disrupting lipid membranes. Immunogold labeling showed that the peptides flow into fungal cells after damaging their membranes. The lack of cyO2 in the vacuoles may be due to a lack of active transport and binding to cytoplasmic proteins. Finding cyO2 in the cytoplasm may indicate that some intracellular targets are affected and partially responsible for the antifungal activity. In addition, singular fungal cells seemed to be completely free of bound cyclotide. The presence of these unaffected cells supports the hypothesis that membrane disruption only occurs when the local cyclotide concentration exceeds a certain threshold (Huang et al., 2009; Burman et al., 2014). The non-permeabilized cells may have slightly different lipid compositions that increase this threshold. Alternatively, the affected cells may have absorbed so much of the cyclotide that the remaining free concentration is below the threshold for the persistent cells.

*Fusarium oxysporum* was unable to break down the structure of cyO2 over a period of multiple days. A common mechanism of fungal pathogenesis involves detoxification of plant antibiotics, i.e., degrading defense molecules (Morrissey and Osbourn, 1999). However, our results show that the antifungal effects of cyclotides persist for extended periods of time. This also means that a potentially substantial nutritional resource (i.e., the amino acids comprising the stable structure of the cyclotides) is unavailable to the pathogen.

We subsequently demonstrated the activity of three cycloviolacins against three bacterial plant pathogens: *P. syringae* pv. *syringae, P. atrosepticum*, and *D. dadantii*. Cycloviolacins (including cyO2, cyO3 and cyO19) have been shown to exhibit assay-dependent broad spectrum antimicrobial activity against human pathogens, with MICs of 10–20 μM (Strömstedt et al., 2017). The MICs against plant pathogens observed in this work varied between 25 and 100 μM. To avoid problems relating to the presence of activity-inhibiting factors in some standard rich growth media, we used M9 minimal media in the antibacterial activity assays. This medium can sustain bacterial growth and is suitable for testing peptides’ antimicrobial activity (Preiter et al., 2005; Czajkowski et al., 2017; Tanui et al., 2017). Such modifications of assay conditions are reasonable if they mimic the natural microenvironment in which the pathogen interacts with plant cells; the plant cell sap released during the process of bacterial infections consists primarily of water, salts, sugars, and organic acids (Evert et al., 2014), so its composition resembles that of the M9 medium. The observed MICs of around 100 μM against *D. dadantii* are relatively high, but the concentration of cyclotides in the cell sap must be accounted for when drawing conclusions about their activity and role in plants. Previous studies showed that as much as 1.5 g of cyclotides could be isolated from 1 kg of wet *V. odorata* plant material (Ireland et al., 2006). Consequently, these peptides are likely to be highly concentrated in the cell sap. Additionally, cyclotides are not evenly distributed across the different parts of the plant, and their concentrations may be much higher in specialized tissues such as the epidermis (Trabi and Craik, 2004; Slazak et al., 2016).

But how is the distribution of different types of cyclotides produced linked to their function in *V. odorata*? Cyclotides obviously have distinct biological activities and roles. For example, the antimicrobial action of cycloviolacins is demonstrated in this work, and kalata B1 is one of many cyclotides shown to be active against herbivorous insect larvae (Jennings et al., 2001; Gruber et al., 2007). We therefore used two complementary imaging techniques, immunohistochemistry and MALDI-MSI, to visualize the distribution of four cyclotides in this plant species. The distributions of cyclotides in flowers and seeds determined by immunohistochemistry support the hypothesis that cycloviolacin cyclotides (cyO2, cyO3, cyO13, cyO19) are components of an antimicrobial defense system; localization in the ovary walls, integuments, and the seed endosperm is characteristic of antifungal compounds (Osbourn et al., 1994; Cooper et al., 1996; Morrissey and Osbourn, 1999; Lay et al., 2003; Nuringtyas et al., 2012). Cyclotides were also present in multiple intracellular bodies in embryos, which is presumably linked to the vacuolar storage of these polypeptides (Conlan et al., 2011; Slazak et al., 2016); non-differentiated meristematic cells contain multiple small vacuoles instead of the single large vacuole observed in vegetative tissues (Seguí-Simarro and Staehelin, 2006). The presence of large quantities of cyclotides in the embryo procambium may partially explain their presence in the phloem as reported by Slazak et al. (2016). In the earlier stages of development, the cells that ultimately become sieve elements have vacuoles. These vacuoles rupture during differentiation (Heo et al., 2014), potentially releasing the cyclotides into the phloem sap.

Although immunohistochemistry offers high resolutions, especially when combined with immunogold and TEM methods, it cannot yet be used to characterize the distribution of kB1 and Möbius cyclotides because antibodies raised against cyO2 do not bind to these peptides (Uddin et al., 2017). Moreover, kB1 is reportedly washed away during tissue preparation for immunohistostaining (Conlan et al., 2011). High resolution MALDI-MSI is an alternative imaging technique capable of detecting all cyclotides that ionize and are present in sufficiently high concentrations. However, the preparation of plant samples for MALDI-MSI is challenging; it requires embedment, cryosectioning, thaw mounting and drying of sections on glass slides. Plant tissues are fragile and prone to shrinkage when cryosectioned and dried due to their high water content and the presence of the cuticle, cell walls, and air-filled intercellular spaces in the tissues (Dong et al., 2016). The method of plant tissue processing for MALDI-MSI that we developed in this work thus constitutes an important advancement. Only minor shrinkage of leaf cross sections occurred; this behavior is common and has been reported previously (Dong et al., 2016).

The association of kB1 with ground tissues such as the petiole parenchyma, root cortex and leaf mesophyll is something that might be expected of a defense chemical active against insect herbivores. An insect feeding on whole plant organs will always ingest defense compounds present in the mesophyll or petiole ground tissues (Nuringtyas et al., 2012). This distribution may also be relevant in interactions with other herbivorous pests such as spider mites, which feed directly on the mesophyll by piercing the epidermis (Bensoussan et al., 2016). The distribution of cyO2, cyO3, and cyO19 revealed by MALDI-MSI confirmed our previously reported immunohistochemical findings (Slazak et al., 2016). However, MALDI-MSI made it possible to determine the distributions of individual cycloviolacins. More pronounced differences in cyclotide accumulation between tissues were observed in an earlier immunohistochemical study (Slazak et al., 2016) because the antibodies used in the earlier work are specific to all three cyclotides and the peptides co-localize. The localization of the peptides reported here and in earlier publications strongly suggests that they play a role in plant antimicrobial defense, particularly against pathogenic fungi. The epidermis and its cuticle, cell walls, and antibiotic chemicals constitute a plant’s first line of defense against pathogens, which must cross this barrier to invade the plant (Knogge, 1996). It appears that the ground tissues surrounding the vascular bundle serve as a secondary chemical bulwark. The collenchyma and pericycle (in the petiole and root, respectively) are rich in cyclotides, and protect the vascular tissues that conduct the plant’s nutrients and assimilates. As such, they serve as defenses against fungi like *F. oxysporum.* that spread throughout the plant *via* the vascular bundles (Dean et al., 2012).

## Conclusion

This work presents an in-depth analysis of cycloviolacin cyclotides (cyO2, cyO3, cyO13, and cyO19) as part of antifungal and antibacterial defense system in *V. odorata*. The activity against plant pathogens is shown as well as the antifungal mechanism of action. Finally, we link the tissue and organ distributions of cyclotides with different biological activities to their presumed biological roles – defense against microorganisms and plant pests.

## Author Contributions

BS planned and designed the research, drafted the manuscript, and participated in all the experiments. MKa participated in immunohistochemistry and immunogold experiments. AAS performed liposome leakage assays. AS participated in immunohistochemistry experiments. MKr performed antibacterial assays. MS and PA participated in MALDI-MSI experiments. JB participated in immunohistochemistry and immunogold experiments. EK participated in immunohistochemistry experiments and isolation of tissues for extraction. UG supervised the study and participated in mass spectrometry experiments. All the authors participated in revising and finalizing the manuscript.

## Conflict of Interest Statement

The authors declare that the research was conducted in the absence of any commercial or financial relationships that could be construed as a potential conflict of interest.
